# Supplementary material for: Cultured Mesenchymal Cells from Nasal Turbinate as a Cellular Model of the Neurodevelopmental Component of Schizophrenia Etiology
Source: Int J Mol Sci. 2023 Oct 19;24(20):15339. doi: 10.3390/ijms242015339 (PMC10607243; doi:10.3390/ijms242015339)
Supplement: Supplementary file 1 [file ijms-24-15339-s001.zip › Supplemental Table S1.pdf]

Supplemental Table S1. Expression of mesenchymal markers in the MC cluster (MT-CTRL), CNON (CNON-CTRL) and cluster 9 of embryonic brain (CS14_3, (19)) and bulk CNON (average Transcripts per Million transcripts from 255 CNON samples). According to the Mesenchymal and Tissue Stem Cell Committee of the International Society for Cellular Therapy [31], "MSC must express CD105, CD73 and CD90, and lack expression of CD45, CD34, CD14 or CD11b, CD79a or CD19 and HLA-DR surface molecules". Other prominent markers of mesenchymal cells are also included in the table.

	Gene	Percentage of Cells Expressing gene in the MC cluster	Percentage of Cells Expressing gene in CNON-CTRL	Percentage of Cells Expressing gene in cluster 9, CS14_3	Bulk CNON, TPM
Must be expressed	NT5E (CD73)	7.20	68.8	32.62	299.07
	THY1 (CD90)	47.88	100	20.92	1347.66
	ENG (CD105)	10.59	77	25.89	16.52
Must NOT be expressed	CD34	0	1.64	1.06	1.12
	PTPRC (CD45)	2.54	0.43	0	0.59
	HLA-DRA	49.58	0.07	0.35	0.10
	HLA-DRB1	44.07	0.07	0.35	0.03
	HLA-DRB5	0	0	0	0.01
One of them must NOT be expressed	CD14	4.66	32.17	0	9.00
	ITGAM (CD11b)	0.42	0	0	0.06
One of them must NOT be expressed	CD79A	0.42	0.70	0	0.16
	CD19	0	0.02	0	0.04
Other mesenchymal markers	CD44	17.37	98.2	15.96	2296.28
	VIM	99.15	100	100	3869.50
	ALCAM(CD166)	19.49	90.3	29.08	555.48
	HSPA8 (STRO-1)	82.63	99.4	52.13	495.22
	ANPEP (CD13)	2.966	59.0	5.67	125.92
	LUM	36.86	11.4	12.77	87.47
