# Supplementary material for: Cultured Mesenchymal Cells from Nasal Turbinate as a Cellular Model of the Neurodevelopmental Component of Schizophrenia Etiology
Source: Int J Mol Sci. 2023 Oct 19;24(20):15339. doi: 10.3390/ijms242015339 (PMC10607243; doi:10.3390/ijms242015339)
Supplement: Supplementary file 1 [file ijms-24-15339-s001.zip › Supplemental Table S2.pdf]

Supplemental Table S2. Expression of Wnt- and Notch- pathways genes in CNON (SEP036), the MC cluster of MT (SEP310), cluster 9 (CS14_3) of embryonic brain and bulk CNON (average Transcripts per Million transcripts from 255 CNON samples).

	Gene	Percentage of Cells ExpressingGene in CNON-CTRL	Percentage of Cells Expressing Gene in the MC cluster of MT-CTRL	Percentage of Cells Expressing Gene in Cluster 9 (CS14_3)	Bulk CNON, TPM
Notch pathway	NOTCH1	3.96	16.10	4.96	1.21
	NOTCH2	45.98	11.02	15.60	96.47
	NOTCH3	4.66	16.10	34.04	2.22
	JAG1	14.09	28.39	53.55	11.15
	PSEN1	19.13	5.08	3.9	85.09
	PSEN2	10.89	3.81	2.13	11.30
	PSENEN	32.52	5.51	17.73	23.58
	APH1A	45.28	11.86	19.86	140.18
	ADAM17	27.96	5.51	4.26	48.05
	HES1	7.52	32.63	22.7	2.15
WNT pathway	WNT3	2.337	2.12	0.35	5.65
	WNT5A	39.99	21.61	0.35	185.16
	WNT5B	67.39	2.12	7.8	150.35
	WNT6	0	4.66	0	0.16
	ROR2	7.77	7.2	0	3.44
	LRP5	20.9	4.24	2.13	7.60
	LRP6	21.6	16.1	3.9	18.00
	AXIN1	7.34	2.12	2.48	9.21
	AXIN2	1.01	6.36	0	1.46
	FZD1	6.89	6.36	2.13	5.82
	FZD2	45.75	9.32	8.16	23.82
	FZD3	1.78	1.27	1.06	0.38
	FZD4	6.12	4.24	1.77	3.04
	FZD5	4.15	3.81	0	1.08
	FZD6	8.37	1.69	1.06	33.64
	FZD7	16.54	6.36	11.7	17.72
	FZD8	1.99	1.69	0.71	3.96
	CTNNB1	46.28	18.64	16.31	308.46
	SFRP1	93.86	44.49	1.42	241.89
	SFRP2	82.61	59.32	0.71	27.51
