# Supplementary material for: Cultured Mesenchymal Cells from Nasal Turbinate as a Cellular Model of the Neurodevelopmental Component of Schizophrenia Etiology
Source: Int J Mol Sci. 2023 Oct 19;24(20):15339. doi: 10.3390/ijms242015339 (PMC10607243; doi:10.3390/ijms242015339)
Supplement: Supplementary file 1 [file ijms-24-15339-s001.zip › Supplemental Table S3.pdf]

Supplemental Table S3. Gene ontology enrichment analysis of genes differentially expressed in Mesenchymal cells cluster (adjusted P-value < 0.05) in comparison to all other cell types in the middle turbinate.

GO biological process complete	**fold Enrichment**	**P-value**	**P.adjust**
regulation of neuron projection development (GO:0010975)	5.21	1.59E-04	1.84E-02
neuron projection development (GO:0031175)	3.86	5.52E-04	4.16E-02
neurogenesis (GO:0022008)	3.6	6.93E-06	2.13E-03
neuron development (GO:0048666)	3.44	6.43E-04	4.63E-02
generation of neurons (GO:0048699)	3.32	1.28E-04	1.57E-02
neuron differentiation (GO:0030182)	3.27	2.80E-04	2.59E-02
cell-matrix adhesion (GO:0007160)	10.74	1.21E-04	1.50E-02
cell-substrate adhesion (GO:0031589)	8.97	6.48E-05	1.02E-02
negative regulation of cell adhesion (GO:0007162)	7	7.16E-05	1.05E-02
cell adhesion (GO:0007155)	5.39	4.06E-09	7.96E-06
regulation of cell adhesion (GO:0030155)	4.04	8.41E-05	1.16E-02
tissue migration (GO:0090130)	10.36	6.86E-04	4.89E-02
ameboidal-type cell migration (GO:0001667)	8.66	7.84E-05	1.10E-02
negative regulation of cell migration (GO:0030336)	7.05	6.86E-05	1.02E-02
positive regulation of cell migration (GO:0030335)	5.45	1.56E-05	3.45E-03
cell migration (GO:0016477)	4.82	4.12E-07	2.81E-04
regulation of cell migration (GO:0030334)	4.37	3.28E-06	1.25E-03
